# The principle of *ne bis in idem* and the European arrest warrant as vehicles for the CJEU for redefining the powers of national prosecutions in EU law

**DOI:** 10.3389/fsoc.2025.1546825

**Published:** 2025-02-26

**Authors:** Balázs József Gellér

**Affiliations:** Eötvös Loránd University, Budapest, Hungary

**Keywords:** EU criminal law, CJEU, European arrest warrant, *ne bis in idem*, *res iudicata*, prosecution, judicial authority, effective judicial protection

## Abstract

The article examines how the Court of Justice of the European Union (CJEU) through its judgments enlarged the ambit of the *ne bis in idem* principle in a certain aspect and redefined attributes of national prosecutions. More poignantly, how the Court’s recognition of a possible *res iudicata* effect of prosecutorial decisions binding on other EU Member States triggering *ne bis in idem* protection not only empowered national prosecutions but also implicitly pushed Member States to further harmonize their criminal justice systems. At the same time, by finding that prosecutions may not only issue European arrest warrants (EAW) but also terminate criminal proceedings with *res iudicata* consequences obligatory for other Member States initiating EU wide recognition of the termination’s *ne bis in idem* force, the CJEU elevated national prosecutions—depending on domestic legal prerequisites—to the level of national courts. It is argued that by reading these judgments together, the CJEU’s jurisprudence might have created frictions between the Member States’ criminal justice systems and reshaped terms such as “judicial authority” and “effective judicial protection” in a way which lowers the level of protection afforded to the individual, be that, to make cooperation in criminal matters more effective between Member States.

## 1 Materials and methods

The research employs the general methodology of legal analysis; more specifically, it looks at fundamental principles of law and legal institutions by examining the relevant case-law of the CJEU. In the course of this, it was necessary to explore certain national laws of Member States to understand why the Luxembourg Court reached different conclusions. The study tried to find similarities between the utilization of the *ne bis in idem* principle and cases concerning the EAW by searching for their effect on the powers of national prosecutions as a common dominator. By studying the thus selected jurisprudence, it strives to understand the CJEU’s aspirations and motivations by also pointing out some secondary, possibly adverse consequences.

## 2 Introduction—the *ne bis in idem* principle

It was a long road for the *ne bis in idem* (double jeopardy) principle to be accepted as a human right, protecting EU citizens in cross Member State relations (Van Bockel and Bastiaan, 2010; Hecker, 2015; Rossi-Maccanico, 2021), as opposed to its application only within one jurisdiction already guaranteed by the European Convention on Human Rights (ECHR) Additional Protocol 7 (Bartsch, 2002; Gellér et al., 2002; Gellér, 2004; Turmo, 2020). It must be pointed out that the double jeopardy ban also applies to non-EU citizens and blocks extradition to third countries (Wahl, 2022) and by that EU law does not only override public international law from time to time but also transforms *ne bis in idem* into an international human right as opposed to a limited internal and regional applicability (Van Bockel, 2016; Bárd et al., 2023).

The Framework Decision on the European Arrest Warrant (FDEAW)^1^ adopted in 2002 was the first concrete measure putting the principle of mutual recognition (Janssens, 2013; Willems, 2019), that is, one Member State must recognize the decisions of another Member State’s judicial authorities, into practice (Satzger, 2018; Maffei, 2019). Similarly, the objective of Article 54 of the Convention Implementing the Schengen Agreement (CISA) is to ensure that no one is prosecuted for the same act in several Contracting States on account of this person having exercised his/her right to freedom of movement (Joined Cases C-187/01 Hüseyin Gözütok and C-385/01 Klaus Brügge, para. 38. Case C-469/03. para. 32). For many years, the CJEU widened the scope of application of the *ne bis in idem* principle and also strengthened the EAW and, thereby, the reach of mutual recognition (Ronsfeld, 2023). With the adoption the EU Charter of Fundamental Rights Article 50 of which also defines the protection of *ne bis in idem*, this principle received the highest possible recognition in EU law.^2^

## 3 The public prosecutor’s office as an authority making final decisions in the judgments of the CJEU

### 3.1 The relevant case-law of the CJEU until 2018

The Case C-268/17 AY and the Hungarian legal framework is a good starting point when analyzing the *res iudicata* nature of decisions made by the public prosecutor’s office in EU Member States—particularly decisions to terminate proceedings—and their effect on *ne bis in idem* from an EU law point of view (Gellér, 2024).

Article XXVIII paragraph (6) of the Hungarian Fundamental Law states that “*With the exception of extraordinary cases of legal remedy laid down in an Act, no one shall be prosecuted or convicted for a criminal offence for which he or she has already been finally acquitted or convicted in Hungary or, within the scope specified in an international treaty and a legal act of the European Union, in another State, as provided for by an Act.*”^3^ This definition primarily indicates that the principle is only applicable in the context of ordinary judicial proceedings; however, in the case of retrial or an extraordinary appeal, the principle cannot be applied, which can be seen as a significant—yet legally justified—limitation of the finality (irrevocability) of a decision (Ambrus, 2019, p. 123).

Pursuant to Section 4 (7) of Act XC of 2017 on the Criminal Procedure Code (CPC): “*A criminal proceeding may not be launched or a criminal proceeding already launched shall be terminated, if the act of the perpetrator has already been adjudicated with final and binding effect in a Member State of the European Union; or if a decision was adopted in a Member State regarding the merits of the act which prevents the launch of a new criminal proceeding regarding the same act, pursuant to the laws of the country where the decision was adopted, or the continuation of the criminal proceeding ex officio or based on any ordinary legal remedy.*”^4^

In EU law, as mentioned above, the principle of *ne bis in idem* is enshrined in Article 54 of the CISA,^5^ Article 50 of the Charter,^6^ point 2 of Article 3^7^, and point 3 of Article 4^8^ of the FDEAW (Coffey, 2023).

In one of the earliest CJEU judgments on the *ne bis in idem* principle—in the Joined Cases C-187/01 Hüseyin Gözütok and C-385/01 Klaus Brüggem—the Court had to decide whether the decision to terminate the criminal proceedings by the public prosecutor could be considered a “*final judgment*” as specified in Article 54 of the CISA. The Court ruled in para. 48 that “*the ne bis in idem principle laid down in Article 54 […] also applies to procedures whereby further prosecution is barred, such as the procedures at issue in the main actions, by which the Public Prosecutor in a Member State discontinues, without the involvement of a court, a prosecution brought in that State once the accused has fulfilled certain obligations and, in particular, has paid a certain sum of money determined by the Public Prosecutor.*”

The *Gözütok and Brügge* judgment was groundbreaking in many ways (Rosanò, 2017; Mancano, 2023). It not only enlarged the ambit of *ne bis in idem* protection through a *contra legem* interpretation of the concept “final judgment”—which a prosecutorial decision clearly is not—but it also strengthened mutual recognition encompassing now other decisions than court judgments, and as a secondary effect, it also forced Members States to harmonize further their criminal justice systems. This latter consequence was a result of the realization on part of the Member States that if they do not want to recognize a decision completely alien to their own jurisdictions, they have to adopt solutions and institutions similar to the other Member States.

It was obvious, however, that Member States will not give up easily the core of their sovereignty, which is their right to create criminal law (*ius puniendi*) (Ambos, 2013), and wish to utilize it according to their own aspirations, and either through a head-on refusal as a State or by implicit and individual institutional sabotage will try to cling on to their powers related to criminal justice as much as possible.^9^

The CJEU has a difficult task when interpreting the above cited Articles defining *ne bis in idem*, although it already held in para. 40 of the Case C-261/09 Gaetano Mantello that: “*In view of the shared objective of Article 54 of the CISA and Article 3(2) of the [FDEAW], which is to ensure that a person is not prosecuted or tried more than once in respect of the same acts, it must be accepted that an interpretation of that concept given in the context of the [CISA] is equally valid for the purposes of the [FDEAW].*” The Court thus has to be cognizant of the fact that Member States may either intentionally or subconsciously protect their citizens and simultaneously not put enough effort into protecting citizens of other Member States.

Therefore, the CJEU in the operative part of Case C-491/07 Turansky developed a fundamental test further clarifying when a prosecutorial or even a decision by the investigative authority will trigger *ne bis in idem* protection: “*the* ne bis in idem *principle enshrined in Article 54 […] does not fall to be applied to a decision by which an authority of a Contracting State, after examining the merits of the case brought before it, makes an order, at a stage before the charging of a person suspected of a crime, suspending the criminal proceedings, where the suspension decision does not, under the national law of that State, definitively bar further prosecution and therefore does not preclude new criminal proceedings, in respect of the same acts, in that State.”* It is evident that the decisive factor is not the nature of the authority issuing the decision to terminate or suspend the proceedings but rather whether the decision possesses *res iudicata* effect under the national law of the Member State in question.^10^ In other words, the judgment indicates that the determination of the *res iudicata* character of decisions made by the public prosecutor falls within the competence of Member States. Furthermore, it is noteworthy that the CJEU emphasized this in the context of decisions arising from procedures involving a substantive investigation of the offense.

The CJEU, while giving thus wide discretion to Member States to assign *res iudicata* effect to their prosecutorial or even investigative authority decisions and by that compelling the other Member States to accept and honor these, it tried to exclude decisions which were factually baseless. Such decisions are not necessarily the results of sham procedures but still lack objective grounds on which any decision must be founded. Hence in the operative part of Case C-486/14 Kossowski (2016), in which there was no detailed investigation by the Polish police, the CJEU confirmed that “*the principle of* ne bis in idem *laid down in Article 54 of the Convention signed in Schengen (Luxembourg), read in the light of Article 50 of the Charter, must be interpreted as meaning that a decision of the public prosecutor terminating criminal proceedings and finally closing the investigation procedure against a person, albeit with the possibility of its being reopened or annulled, without any penalties having been imposed, cannot be characterised as a final decision for the purposes of those articles when it is clear from the statement of reasons for that decision that the procedure was closed without a detailed investigation having been carried out; in that regard, the fact that neither the victim nor a potential witness was interviewed is an indication that no such investigation took place.*”

Consequently, it seemed that by 2018, a prosecutorial termination of criminal proceedings activated *ne bis in idem* protection, if there was a detailed investigation (Kossowski test) and the termination (suspension) under the national law of the executing State, definitively bared further prosecution in respect of the same acts against the same person (Turansky test).

### 3.2 Some relevant issues arising in Case C-268/17 AY (2018)

The *AY* case represents a significant turning point or at least an important further specification of the requirements for affording *ne bis in idem* (*res iudicata*) effect to non-judicial decision under EU law in criminal proceedings.

The so-called “*Sanader and H*” (H is AY in the case before the CJEU) case is unique in EU legal history and might also be singular worldwide since it involves a former prime minister of a Member State (Croatia), and H, the CEO of the largest than state-controlled (through shares) and partially owned firm (MOL) of a Member State (Hungary), both were indicted for bribery in another Member State (Croatia). Moreover, the case was adjudicated by two respected international arbitration tribunals, the ICSID and the UNCITRAL (Gellér, 2024, p. 73–74).

The legally most important facts of the case are the following (Gellér, 2024, p. 72–74). In 2009, the Croatian Special Prosecution (CSP) started an investigation into the question of whether the contracts between MOL and Croatia concerning ownership of INA (the Croatian oil company) were harmful to Croatia. In 2011, CPS requested that Hungary should hear H as a suspect in the case involving bribery. Hungary, however, refused the request, relying on traditional cooperation in criminal matters, and at the same time, the Hungarian Central Chief Prosecution (HCCP) started an investigation on its own, based on the same facts (13 September 2011). On 20 December 2012, the HCCP terminated the case after a detailed investigation, stating that no crime had been committed. H was never charged, only interviewed as a witness. Using the possibility under Hungarian law of bringing private prosecutions, a small shareholder of MOL brought a private indictment against H for fraud, embezzlement, and bribery founded again on the same facts (6 September 2013). In the meantime, the Zagreb County Court in Croatia (ZCC) issued an EAW against H, which was not executed by the Budapest High Court (BHC) as the Court cited *ne bis in idem* based on the termination of the proceedings in Hungary by prosecution.^11^ Nevertheless, the trial following the private prosecution initiated by the small shareholder continued in Hungary, and on 26 April 2014, the BHC acquitted H of the fraud and embezzlement charges and terminated the case regarding bribery, stating that the small shareholder did not have standing to bring a private prosecution for bribery. Subsequently, on appeal, the Capital Court of Appeals in Budapest (CCA) held that the small shareholder did not have standing as regards bribery. In addition, fraud and embezzlement would not be punishable if bribery had been committed since H would not have been expected to report the illegal use of the funds (that is, the alleged bribing of Sanader). Holding otherwise would violate the right against self-incrimination (3 December 2014). On 15 December 2015, the second Croatian EAW was sent to Hungary, but the BHC did not examine it, claiming that there are no new facts; similar treatment was given to the third EAW which was submitted to the Hungarian authorities by the ZCC on 27 January 2017.

A request for a preliminary ruling under Article 267 TFEU from the ZCC was received by the CJEU on 18 May 2017. It concerned the interpretation of Article 1(2), Article 3(2), and Article 4(3) of FDEAW concerning the issuing of an EAW by the ZCC in the proceedings against AY.

The CJEU gave a two-pronged answer to the ZCC’s five questions out of which only the second concerns our topic. According to the second point of the operative part of the judgment *“Article 3(2) and Article 4(3) of [FDEAW] must be interpreted as meaning that a decision of the Public Prosecutor’s Office, such as that of the Hungarian National Bureau of Investigation in question in the main proceedings, which terminated an investigation opened against an unknown person, during which the person who is the subject of the European arrest warrant was interviewed as a witness only, without criminal proceedings having been brought against that person and where the decision was not taken in respect of that person, cannot be relied on for the purpose of refusing to execute that European arrest warrant pursuant to either of those provisions.”*

Although this judgment could be seen as further detailing the conditions for non-judicial *ne bis in idem*, it can be argued also that there is a significant flaw in this decision, namely, that it fails to address the differing evidentiary requirements in various Member States for charging a person. Consequently, if less evidence is needed for example in Germany to charge somebody as is necessary in Austria or Hungary, then *ne bis in idem* protection is afforded to persons much earlier and based on a lesser level of proof in Germany than in the other two Member States.

Accordingly, while the *Turansky* judgment links the *res iudicata* effect of prosecutorial decisions to national law, the *AY* judgment imposes an additional condition, specifically that such an effect is only admissible when the decision is ending proceedings in which the person in question was charged. This approach creates disparities in the EU wide application of *ne bis in idem* as the criminal procedure laws of Member States mandate charging a person with a crime under varying conditions, at different procedural stages, and with differing evidentiary thresholds. This will cause greater legal inequality and uncertainty among Member States and especially persons involved in competing criminal proceedings in different Member States. At the same time, the argument for the correctness of the CJEU judgment is clear and easy to accept: *ne bis in idem* can be invoked only by the same person, who has been investigated or prosecuted for the same facts before. A witness, although possibly a person of interest, has not yet been charged; therefore, it cannot be said that he/she was subjected to criminal proceedings.

It was even more striking that in Hungary, the prosecution and the courts themselves had differing opinions on the *res iudicata* quality of their decisions. The Hungarian Supreme Court (Kuria) did not wait for long after the *AY* verdict to voice its opinion on this matter. Its judgment published as decision No. BH 2018.301^12^ stated that the prohibition of double prosecution—the application of the *ne bis in idem* principle—is a constitutional requirement in Hungary, enshrined in the Fundamental Law. The presumption of innocence under the Fundamental Law explicitly and exclusively links the final determination of criminal liability to a court’s final decision, excluding decisions by other authorities. Furthermore, the provision enshrining the *ne bis in idem* principle applies explicitly to acquittals and convictions, which, by definition, can only be issued by a court. Consequently, within the Hungarian legal framework, only a court decision can possess *res iudicata* effect under the Fundamental Law. However, this does not preclude the recognition of legal acts issued by other authorities of other Member States which have equivalent effects.^13^

The Kuria decision under discussion raises many questions. Foremost, it seems that according to it, all prior decisions rendered by courts or other authorities which endowed *res iudicata* force to prosecutorial decisions were unconstitutional. Furthermore, it could be argued that it is the Constitutional Court of Hungary and not the Kuria which is competent in adjudicating constitutional matters. In addition, the absence of the *res iudicata* effect of prosecutorial decisions terminating criminal proceedings raises several new questions. First, it introduces legal uncertainty as there is no clear justification for why a subsequent prosecutorial decision can override an earlier one. Second, the statute of limitations offers insufficient safeguards against potential state abuse, which could leave an individual under the prolonged threat of criminal proceedings for years. Third, the availability of substitute private prosecution already provides adequate protection for victims in case the victims feel that the conclusion of the criminal proceedings by the prosecution was not satisfactory.

Moreover, according to the Kuria, a prosecutor’s decision to terminate proceedings is less favorable to the accused than an acquittal since within Hungary a termination by the prosecution is now not final whereas an acquittal will entail a *ne bis in idem* protection. This approach effectively compels an innocent individual to seek an indictment in the hope of securing an acquittal as only the latter guarantees immunity from future proceedings. This inconsistency highlights the tension not only between different approaches within national jurisdictions (which may change by time as well) but also draws attention to discrepancies between national constitutional principles and evolving EU jurisprudence.

## 4 Evolution or further confusion?

Why would the CJEU allow a Member State to deprive someone of *ne bis in idem* protection by simply not charging that person? It could be argued that this is because of the term “criminal charge” in Article 6 para. 1 of the ECHR.^14^ Indeed, criminal charge and hence criminal proceedings have an autonomous conventional meaning,^15^ and the European Court of Human Rights (ECoHR) has developed two tests for assessing whether proceedings are criminal in their nature.^16^ The idea behind the autonomous concepts, however, is precisely that states should not be able, by simple labeling, to avoid adherence to the Convention. Similarly, by attaching the *ne bis in idem* protection to the condition of charging a person, Member States may withhold such protection, where other Member States, based on a different standard of proof, by charging the person may afford him/her said right, and this clearly leads to discriminatory treatment, as argued above. In addition, it could be said that too early a charge is in violation of the directive on the presumption of innocence.^17^ It is, therefore, suggested that common grounds must be developed within EU law as to the standard of proof and evidence required to charge a person within a Member State (Gellér, 2024, p. 77).

It is interesting to observe that the CJEU arrived at a very different result in Case C-147/22 *Terhelt5* (2023) involving Hungary and Austria. This case concerned suspected bribes to public agents through several companies established in different Member States to influence the decision to be taken in a procedure for the award of a public contract in Hungary. The Hungarian person of interest was not interviewed as a suspect in the context of the investigation carried out by the Central Public Prosecutor’s Office for the Prosecution of Financial Crimes and Corruption in Austria (WKStA) as the investigative measures taken by the public prosecutor’s office on 26 May 2014 seeking to locate him proved unsuccessful (para. 17. of Case C-147/22). By order of 3 November 2014, the WKStA discontinued the pre-trial investigation without further action, taking the view—referring to the results of the investigations carried out up to that point by the Austrian, British, and Hungarian authorities—that there were no real grounds for continuing the criminal proceedings, within the meaning of para. 190(2) of the Austrian Code of Criminal Procedure (StPO). The public prosecutor’s office considered that, since there was no evidence that one of the suspects and the accused had actually committed the acts of corruption referred to in para. 307(1)(6) of the Austrian Criminal Code (StGB), those acts had not been established with sufficient certainty to give rise to a criminal conviction, with the result that it was necessary to discontinue the proceedings (para. 18. of Case C-147/22). On 10 April and 29 August 2019, the National Public Prosecutor’s Office in Hungary filed with the Budapest High Court (BHC)—the referring court—an indictment on the basis of which criminal proceedings were brought in Hungary against the suspect for acts of corruption, within the meaning of paras. 254(1) and (2) of Act IV of 1978 [the Hungarian Criminal Code (HCC)]. The BHC decided to stay the proceedings and to refer the case for preliminary ruling (para. 21. of Case C-147/22).

When reading the facts of this case, it is clear that it involves issues comparable to *Turansky* and *AY*, yet *AY* is not mentioned once in the judgment. Instead of facing the music, the Court decided to sweep the problem under the carpet as I pointed out in another study (Gellér, 2024, p. 82). The Court found that the fact that the suspect was not questioned was not hindering *ne bis in idem* protection, nor indeed did the fact that the decision of the prosecution had no *res iudicata* effect under national law, although proceedings could only be continued under strictly defined conditions, such as the emergence of significant new facts or evidence, and provided that, in any event, the offense is not time-barred. The same was the case in *AY* regarding decisions of the Hungarian prosecution, be that the Kuria changed its views on the *res iudicata* power of prosecutorial decisions or at least declared the lack of such after the *AY* judgment.

It is difficult to see why someone who was never formally charged and did not participate in the proceedings could benefit of *ne bis in idem* protection when a lack of evidence led to the termination of the proceedings (*Terhelt5*), while another person who did participate in a detailed investigation and gave a testimony under oath as a witness (clearly being a person of interest) was refused the same protection when the proceedings revealed that no crime had been committed (*AY*) (Gellér, 2024, p. 82).

At first glance, the *AY* judgment looks as a decision whereby the CJEU simply wanted to end the streak of judgments constantly enlarging *ne bis in idem*’s field of application. It added a further condition to the *Turansky test*: For a case to fall under *ne bis in idem,* it is no longer sufficient that the first proceedings be terminated by a decision having *res iudicata* effect under the national law of the Member State: The person in question also has to be formally charged.

The issue at point is raised currently by Case C-701/23 Swiftair. It is clear from the questions formulated by the Tribunal Judiciaire de Paris (France) that the difference between provisional dismissal and a dismissal which constitutes a final disposal of the case under Spanish law, and also the fact that no one was charged, and additionally the possible criminal responsibility of legal persons and the effect of *ne bis in idem* on them is examined. While one awaits the Opinion of the Advocate General with great interest, since this case advances legal points not yet clarified sufficiently by previous case law, it is anticipated that yet again not all problems will be addressed (Gellér, 2024, p. 84).

## 5 The public prosecutor’s office as an independent judicial body under EU law

The issue of whether a decision by the prosecution can induce *ne bis in idem* protection through its *res iudicata* character is—or at least should be—connected to another quality of the prosecution, namely, it being a judicial authority. This latter characteristic, if interpreted correctly, must entail the authority’s independence and impartiality within the meaning of Article 6 para. (1) of the ECHR as developed by the ECoHR’s jurisprudence.^18^

The concept of “judicial authority” has been construed by the CJEU as well. Its judgment in Joined Cases C-508/18 and C-82/19 PPU (known as *OG and PI* cases) (2019) involved the following facts^19^: OG, a Lithuanian national residing in Ireland, had his surrender sought under an EAW issued by the Public Prosecutor’s Office in Lübeck for the prosecution of murder and grievous bodily harm allegedly committed in 1995. The *PI* case concerned the request for the surrender of PI, a Romanian national, for the prosecution of a criminal offense identified as organized or armed robbery, pursuant to an EAW issued by the Public Prosecutor’s Office in Zwickau (Germany) in 2018. The High Court in Ireland declared the warrant enforceable, and PI was subsequently arrested based on it. In both cases, the key issue was that the EAWs were not issued by a court but by the public prosecutor’s office, as permitted by German law.

Within the meaning of Article 6(1) of the FDEAW “*The issuing judicial authority shall be the judicial authority of the issuing Member State which is competent to issue a European arrest warrant by virtue of the law of that State*.” In accordance with the principle of procedural autonomy, Member States may designate the “judicial authority” competent to issue an EAW based on their national law, but the meaning and scope of this concept cannot be left completely to the discretion of each Member State.

It is important to note that, although the German public prosecutor’s office is subordinate to the government and the government has the right to issue instructions, the CJEU did not regard the public prosecutor’s office as part of the executive branch solely on this basis.

In German law, Section 146 of the *Gerichtsverfassungsgesetz* (GVG)^20^ provides that “*The officials of the public prosecution office must comply with the official instructions of their superiors.*” Pursuant to Section 147 of the GVG: “*The right of supervision and direction shall lie with: 1.* *the Minister of Justice and Consumer Protection in respect of the Federal Prosecutor General and the federal prosecutors; 2. the Land Department of Justice in respect of all the officials of the public prosecution office of the Land concerned; 3. the highest-ranking official of the public prosecution office at the Higher Regional Courts and the Regional Courts in respect of all the officials of the public prosecution office of the given court’s district*.”^21^

Para. 50 of the judgment in *OG and PI* states that “*the Court has previously held that the words ‘judicial authority’, contained in that provision, are not limited to designating only the judges or courts of a Member State, but must be construed as designating, more broadly, the authorities participating in the administration of criminal justice in that Member State, as distinct from, inter alia, ministries or police services which are part of the executive*.” Pursuant to para. 51, this implies that “*the concept of a ‘judicial authority’, within the meaning of Article 6(1) of Framework Decision 2002/584, is capable of including authorities of a Member State which, although not necessarily judges or courts, participate in the administration of criminal justice in that Member State.*”

Para. 60 of the judgment says that a public prosecutor’s office, which is responsible for prosecuting criminal offenses and bringing suspects before a court, must be considered as participating in the administration of justice of the relevant Member State. While para. 75 adds that when the law of the issuing Member State grants the authority to issue an EAW to a body that is not a court but participates in the administration of justice, the decision to issue the warrant, including its proportionality, must be subject to court proceedings that fully meet the requirements of effective judicial protection. Para. 88 of the judgment stipulates that if public prosecutors’ offices are at risk of executive influence in issuing an EAW, they do not meet the requirement of acting independently, as required to be considered an “issuing judicial authority” under Article 6(1) of the FDEAW. The operative part of the judgment concludes that the concept of an “issuing judicial authority” does not include public prosecutors’ offices exposed to the risk of being directed or instructed by the executive, such as a Minister for Justice, in issuing a European arrest warrant.

On the same day (27 May 2019), another judgment was also rendered by the Court in Case C-509/18 *PF*. The CJEU determined that the Prosecutor General of a Member State (in this instance, Lithuania), who operates independently from the judiciary in an organizational sense and performs prosecutorial functions, and whose status in that Member State ensures independence from the executive in the context of issuing an EAW, qualifies as an issuing judicial authority under the meaning of the FDEAW.

Later that year, in December of 2019, the CJEU examined the French public prosecutor’s office in this respect. In the Joined Cases C-566/19 PPU and C-626/19 PPU (known as *JR and YC* cases), the requests for preliminary ruling were made in the context of the execution of EAWs in Luxembourg and in the Netherlands issued by the *Procureur de la République près le tribunal de grande instance de Lyon* (Public Prosecutor attached to the General Court of First Instance of Lyon (France)) for the purpose of prosecuting JR and by the *Procureur de la République près le tribunal de grande instance de Tours* (Public Prosecutor attached to the General Court of First Instance in Tours (France)) for the purpose of prosecuting YC.

Regarding the position of the public prosecutor’s office, pursuant to Article 64(1) of the French Constitution of 4 October 1958: “*The President of the Republic shall be the guarantor of the independence of the Judicial Authority.*”^22^ Within the meaning of Article 5 of Order No 58–1270 of 22 December 1958 enacting the institutional Act on the status of the judiciary: “*Public prosecutors are under the direction and control of their superiors and the Minister of Justice. They are free to speak at the trial*.”^23^ Book I of the normative part of the *Code de procédure pénale* (French Criminal Procedure Code (FCPC)), titled “*The conduct of criminal policy, prosecution functions and investigations*,” consists of four chapters. Chapter I of Book I of the FCPC, entitled “*The authorities responsible for the conduct of criminal policy, prosecution functions and investigations*,” includes, inter alia, Articles 30, 31, and 36. Article 30 reads as follows: “*The Minister of Justice conducts the criminal policy determined by the Government. He ensures the consistency of its application on the territory of the Republic. To this end, he shall issue general instructions to the public prosecutors. He may not issue any instructions to them in individual cases.*” Article 31 of the FCPC states that: “*The public prosecutor’s office shall conduct prosecutions and enforce the law with due regard for the principle of impartiality by which it is bound.*” Finally, Article 36 of the CPP provides as follows: “*The Attorney-General may order the public prosecutors, by written instructions and placed in the record of the proceedings, to institute or cause to be instituted proceedings or to submit to the competent court such written requisitions as the Attorney-General deems appropriate.*”^24^

Para. 55 of the judgment in *JR and YC* states that in France, public prosecutors independently assess the necessity and proportionality of issuing an EAW, considering both incriminatory and exculpatory evidence, without undue influence from the executive. Para. 56 adds that, while public prosecutors must comply with instructions from hierarchical superiors, the Court’s case-law, particularly in *OG and PI* and *PF* confirms that the requirement of independence prohibits external instructions, especially from the executive, but allows internal instructions within the prosecutor’s office based on its hierarchical structure in individual cases.

However, in addition to the legal status of the prosecutor’s office and its jurisdiction, para. 59 of the judgment examined, as an additional condition, the right to effective judicial protection and its enforcement in the case of an EAW issued by a public prosecutor’s office: “*The European arrest warrant system entails a dual level of protection of procedural rights and fundamental rights which must be enjoyed by the requested person, since, in addition to the judicial protection provided at the first level, at which a national decision, such as a national arrest warrant, is adopted, there is the protection that must be afforded at the second level, at which a European arrest warrant is issued, which may occur, depending on the circumstances, shortly after the adoption of the national judicial decision.*”

The reference to judicial review here and also in para. 75 of *OG and PI* is difficult to understand as it seems to contradict other similar judgments of the CJEU and the principle of double protection (effective judicial protection), as explained in the Case C-241/15 *Niculaie Aurel Bob-Dogi* (2016). Pursuant to Article 8(1)(c) of the FDEAW: “*The European arrest warrant shall contain the following information set out in accordance with the form contained in the Annex: … (c) evidence of an enforceable judgment, an arrest warrant or any other enforceable judicial decision having the same effect, coming within the scope of Articles 1 and 2.*”

As the Court explained in para. 56 of the *Niculaie Aurel Bob-Dogi* case “*The European arrest warrant system therefore entails, in view of the requirement laid down in Article 8(1)(c) of the Framework Decision, a dual level of protection for procedural rights and fundamental rights which must be enjoyed by the requested person, since, in addition to the judicial protection provided at the first level, at which a national judicial decision, such as a national arrest warrant, is adopted, is the protection that must be afforded at the second level, at which a European arrest warrant is issued, which may occur, depending on the circumstances, shortly after the adoption of the national judicial decision.*”

In other words, the issuance of an EAW must be preceded by a national arrest warrant, which has to be issued by a court or—following the logic of the CJEU—by the public prosecutor’s office if this meets the tests defined in the *OG and PI* and the *PF* judgments. However, if the national arrest warrant was issued by the prosecution, then the EAW must be issued by a court or vice-versa.

On this basis, the CJEU stated in the operative part of Case *JR and YC* that “*as regards a measure, such as the issuing of a European arrest warrant, which is capable of impinging on the right to liberty of the person concerned, that protection means that a decision meeting the requirements inherent in effective judicial protection should be adopted, at least, at one of the two levels of that protection*.”

Finally, in explaining the different interpretations of French law the CJEU stated in para. 69 that “*according to the French Government, in the French legal system, the decision to issue a European arrest warrant may, as a procedural step, be the subject of an action for a declaration of invalidity on the basis of Article 170 of the CCP. Such an action, which is available as long as the criminal investigation is ongoing, enables the parties to the proceedings to enforce their rights. If the European arrest warrant is issued in respect of a person who is not yet a party to the proceedings, that person may bring an action for a declaration of invalidity after his actual surrender and appearance before the investigating judge*.” Accordingly, the Court found that the requirement of effective judicial protection “*which must be afforded any person in respect of whom a European arrest warrant is issued in connection with criminal proceedings are fulfilled if, according to the law of the issuing Member State, the conditions for issuing such a warrant, and in particular its proportionality, are subject to judicial review in that Member State*.”

It is worth observing that the CJEU’s jurisprudence by affording, although depending on corresponding national law, *res iudicata* effect to prosecutorial decisions previously reserved for courts (tribunals) and additionally, finding that they may qualify as judicial authority and form part of effective judicial protection clearly departed from the stringent Article 6 (1) ECHR case law of the ECoHR.^25^

## 6 Conclusion

The CJEU chose to supplement EU legislation in the field of criminal justice by forcing Member States to harmonize their respective laws or be compelled to recognize decisions completely foreign to their own systems. The most effective vehicle for that proved to be the *ne bis in idem* principle which not only appears in several key legal instruments but is part of the FDEAW as grounds for refusing the execution of an EAW (Gellér, 2018). By equating prosecutorial decisions as far as terminating criminal proceedings is concerned with court judgments, the CJEU forced Member States to recognize the other Members State’s prosecution’s decisions even where her own prosecution would have not been authorized to render such a decision. This undoubtedly was more expedient, than waiting for the States to bring their law in conformity with each other, but it also presupposed that Member States which reserved these powers for their courts did so unnecessarily and additionally this jurisprudence may have run counter the case law of the ECoHR. This latter problem is an issue to be further examined in the light of para. 168 of Opinion 2/13 of the CJEU of 18 December 2014 (Odermatt, 2015) and Article 53 of the ECHF.^26^

The diversity of national laws and the unjustly diverging protection provided by EU law in different Member States—and the emphasis is on EU law affording varying levels of rights protection to EU citizens—is keenly exhibited by the tests which make *ne bis in idem* protection under EU law dependent on the charging of the individual under national law which lacks any EU standard.

In addition, the CJEU also empowered prosecutions—subject to appropriate national legislation—to issue EAWs which must be executed by other Member States. The justification of these judgments redefines judicial authority and effective judicial protection encompassing prosecutorial decisions as well. Undoubtedly, the eagerness of the CJEU to draw the criminal justice systems of Member States closer and closer overrode not only the prior understanding of these concepts as parts of a larger human rights protection system but implies an evaluation of the affected Member States’ prosecutorial system by which an independent prosecution is arguably more in conformity with EU expectations than one under governmental supervision.

One understands the frustration of those striving for an ever-closer cooperation between Member States with tardy EU law-making caused by Member State objections; nevertheless, judicial activism on part of the CJEU must not create inhomogeneous EU law protection in different Member States and must not chip away on rights developed and cherished for decades.

## Data Availability

The original contributions presented in the study are included in the article/supplementary material, further inquiries can be directed to the corresponding author.

## Footnotes

^1^Council Framework Decision 2002/584/JHA of 13 June 2002 on the European arrest warrant and the surrender procedures between Member States (FDEAW) [2002] OJ L 190/1.

^2^“*Right not to be tried or punished twice in criminal proceedings for the same criminal offence:* *No one shall be liable to be tried or punished again in criminal proceedings for an offence for which he or she has already been finally acquitted or convicted within the Union in accordance with the law.*”

^3^The official English version of the Fundamental Law is available at: https://njt.hu/jogszabaly/en/2011-4301-02-00.

^4^The official English version of the Code of Criminal Procedure is available at: https://njt.hu/jogszabaly/en/2017-90-00-00.

^5^Article 54 of the Convention implementing the Schengen Agreement of 14 June 1985, signed at Schengen on 19 June 1990: “*A person whose trial has been finally disposed of in one Contracting Party may not be prosecuted in another Contracting Party for the same acts provided that, if a penalty has been imposed, it has been enforced, is actually in the process of being enforced or can no longer be enforced under the laws of the sentencing Contracting Party.*”

^6^“*Right not to be tried or punished twice in criminal proceedings for the same criminal offence:* *No one shall be liable to be tried or punished again in criminal proceedings for an offence for which he or she has already been finally acquitted or convicted within the Union in accordance with the law.*”

^7^“*Grounds for mandatory non-execution of the European arrest warrant,—“if the executing judicial authority is informed that the requested person has been finally judged by a Member State in respect of the same acts provided that, where there has been sentence, the sentence has been served or is currently being served or may no longer be executed under the law of the sentencing Member State.*”

^8^“*The executing judicial authority may refuse to execute the European arrest warrant:—“where the judicial authorities of the executing Member State have decided either not to prosecute for the offence on which the European arrest warrant is based or to halt proceedings, or where a final judgment has been passed upon the requested person in a Member State, in respect of the same acts, which prevents further proceedings.*”

^9^It is noticeable that even in the USA states will not relinquish their control over criminal justice. Approximately 5–10% of criminal cases fall under federal and 90–95% under state jurisdiction. In 2022, state courts processed approximately 15.6 million criminal cases, including 11.5 million misdemeanours and 2.9 million felonies. https://www.ncsc.org/newsroom/at-the-center/2024/court-statistics-project-releases-trial-court-caseload-trends?utm_source=chatgpt.com Accessed 12 December 2024. In the fiscal year ending 31 March 2024, U.S. district courts saw a combined total of 414,026 filings for civil cases and criminal defendants. https://www.uscourts.gov/data-news/reports/statistical-reports/federal-judicial-caseload-statistics/federal-judicial-caseload-statistics-2024 Accessed 12 December 2024. Between 1998 and 2022, 3,763,862 persons in federal investigations (https://fccps.bjs.ojp.gov/home.html?dashboard=FJSP-LawEnforcement&tab=LawEnforcementInvestigationsInitiatedAdvanced; Accessed 12 December 2024).

^10^The judgment refers to Contracting State because the case was based on CISA.

^11^The Budapest High Court cited Article 4 (3) of the FDEAW.

^12^BH 2018.11.301: Kúria Bfv. III. 1.788/2017. (Hungarian Supreme Court Decision No. 2018.301.)

^13^Last sentence of the operative part of Hungarian Supreme Court Decision No. 2018.301 referring to Article 6 (3) (d) of CPC and Article XXVIII (2) and (6) of the Fundamental Law.

^14^Article 6 Right to a Fair trial: 1. In the determination of his civil rights and obligations or of any criminal charge against him, everyone is entitled to a fair and public hearing within a reasonable time by an independent and impartial tribunal established by law. Judgment shall be pronounced publicly but the press and public may be excluded from all or part of the trial in the interests of morals, public order, or national security in a democratic society, where the interests of juveniles or the protection of the private life of the parties so require, or to the extent strictly necessary in the opinion of the court in special circumstances where publicity would prejudice the interests of justice.

^15^Guide on Article 6 of the European Convention on Human Rights (Prepared by the Registry, 31 August 2022), 9 onwards <https://www.echr.coe.int/documents/d/echr/guide_art_6_criminal_eng> accessed 1 December 2024; *Blokhin v Russia* App no 47152/06 (ECHR, 23 March 2016) para. 179; *Adolf v Austria* (1982) 4 EHRR 313, para. 30.

^16^For the Engel-test, see *Engel v the Netherlands* App nos 5100/71, 5,101/71, 5,102/71, 5,354/72, and 5370/72 (ECHR, 8 June 1976), paras 82–83; for the Welch-test (art 7), see *Welch v UK* App no 17440/90 (ECHR, 9 February 1995); see, for comparison, *A v the Netherlands* App no 29094/09 (5 July 2016).

^17^Directive (EU) 2016/343 of the European Parliament and of the Council of 9 March 2016 on the strengthening of certain aspects of the presumption of innocence and of the right to be present at the trial in criminal proceedings.

^18^“*Die Beamten der Staatsanwaltschaft haben den dienstlichen Anweisungen ihres Vorgesetzten nachzukommen*.” Neugefasst durch B. v. 09.05.1975 BGBl. I S. 1077; zuletzt geändert durch Artikel 1 G. v. 07.10.2024 BGBl. 2024 I Nr. 302. Geltung ab 01.01.1975; FNA: 300–2 Gerichtsverfassung.

^19^The judgement was passed on 27 May 2019.

^20^Gerichtsverfassungsgesetz in der Fassung der Bekanntmachung vom 9. Mai 1975 (BGBl. I S. 1077), das zuletzt durch Artikel 1 des Gesetzes vom 7. Oktober 2024 (BGBl. 2024 I Nr. 302) geändert worden ist.

^21^§ 147 GVG: “*Das Recht der Aufsicht und Leitung steht zu: 1. dem Bundesminister der Justiz und für Verbraucherschutz hinsichtlich des Generalbundesanwalts und der Bundesanwälte; 2. der Landesjustizverwaltung hinsichtlich aller staatsanwaltschaftlichen Beamten des betreffenden Landes; 3. dem ersten Beamten der Staatsanwaltschaft bei den Oberlandesgerichten und den Landgerichten hinsichtlich aller Beamten der Staatsanwaltschaft ihres Bezirks*.”

^22^Accessed https://www.conseil-constitutionnel.fr/en/constitution-of-4-october-1958.

^23^Order No 58–1270 of 22 December 1958 Enacting the Institutional Act on the Status of the Judiciary (*ordonnance n° 58–1,270 du 22 décembre 1958 portant loi organique relative au statut de la magistrature*) https://www.legifrance.gouv.fr/loda/id/JORFTEXT000000339259?utm_source=chatgpt.com Accessed 12 December 2024.

^24^The translation used in the judgment has been quoted rather than the translation of the FCPC found at https://legislationline.org/sites/default/files/2023-10/France_Code_of_criminal_procedure_EN.pdf.

^25^ECoHR Öztürk v. Germany judgment, 1984, § 56, ECoHR Josseaume v. France judgment, 2012, § 32, ECoHR Célice v. France judgment, 2012, § 34, https://ks.echr.coe.int/documents/d/echr-ks/guide_art_6_criminal_eng.

^26^Article 53 Level of protection (The Charter of Fundamental Rights of the European Union). Nothing in this Charter shall be interpreted as restricting or adversely affecting human rights and fundamental freedoms as recognized, in their respective fields of application, by Union law and international law and by international agreements to which the Union, the Community or all the Member States are party, including the European Convention for the Protection of Human Rights and Fundamental Freedoms, and by the Member States’ constitutions.
